# Employing positive psychology to improve radiation therapy workplace culture

**DOI:** 10.1002/jmrs.321

**Published:** 2019-02-01

**Authors:** Darren Hunter, Caroline Wright, Sue Pearson

**Affiliations:** ^1^ Department of Radiation Oncology Peter MacCallum Cancer Centre Victoria Australia; ^2^ Department of Medical Imaging and Radiation Sciences Monash University Victoria Australia; ^3^ School of Medicine University of Tasmania Tasmania Australia

**Keywords:** Australia, burnout, leadership, positive psychology, radiotherapy, stress

## Abstract

The Australian radiotherapy profession is challenged by job dissatisfaction, stress, burnout and unfavourable attrition. This paper will use psychological models to discuss the confluence of job demands, resources and personal characteristics that contribute to these challenges. Factors contributing to burnout and attrition amongst Australian Radiation Therapists will be explored, and a number of leadership strategies will be introduced to improve workplace culture. These strategies – aligned with positive psychology – seek to address staff engagement, emotional needs, and job stressors.

## Background

Australian radiation therapists (RTs) are responsible for planning and delivering radiotherapy (RT) – primarily for cancer treatment. In the early 2000s, Australia experienced a critical shortage of RTs; impacting on outcomes and quality of life of cancer patients.^1^ The RT shortage coexisted with significant attrition – reflecting increased RT demands and ineffective utilisation of staff skills.^2^ Workforce surveys administered during this period saw the number of RTs qualified for 4–6 years halved from 16.1% of the workforce in 1998, to 8.4% in 2001.^3^

National workforce retention and limited RT positions over the past decade continues to justify concern. Data are scarce on Australian RT retention, however, 2009 projections of *‘extremely high’* attrition and a *‘very tight employment market’* may likely persist.^4^ 2017 data suggested that 20% of RTs were considering leaving their current workplace, and 13% were contemplating departure from the profession.^5^ Dissatisfaction is attributed to perceived monotonous roles/responsibilities, limited progression opportunities, and occupational burnout/distress.^3^, ^6^, ^7^ Broader health research has correlated job dissatisfaction, stress and burnout with compromised employee wellbeing and higher attrition.^8^, ^9^, ^10^, ^11^, ^12^ However, there is a paucity of research to quantify this correlation in the RT context.

The aims of this report are to: (1) discuss contributing factors to elevated stress, dissatisfaction, turnover and burnout amongst Australian RTs; and (2) suggest employment of positive psychology theories to facilitate change.

## Positive Organisational Psychology

Positive Organisational Psychology (POP) applies many concepts arising from positive psychology to the workplace. Originally conceptualised by Martin Seligman^13^ this work promotes key factors that enable individuals to flourish.^14^ As an evidence‐based approach to understanding the individual's potential, POP complements traditional psychology to sit alongside current practice.^15^ POP is effective in fostering a supportive, functional and successful workplace culture.^16^

POP implores individuals to revoke a problem‐focussed workplace in favour of promoting success, strengths and solutions.^16^ Employing the principles of POP to the RT department considers future possibilities, rather than current shortfalls.^15^ Similarly, staff should contemplate necessary improvement initiatives to workplace culture and patient outcomes. Indeed, these initiatives may interrelate; as motivated staff will likely improve service delivery.

Many psychological workplace models used in POP research explore factors contributing to staff satisfaction, health and wellbeing.^8^, ^17^ Two commonly used models include the Job Demands‐Resources (JD‐R) model and the Job Characteristics (JC) model.

### The job demands‐resources (JD‐R) model

The JD‐R model describes stress, health and wellbeing as influenced by opposing dimensions (resources and demands).^8^, ^9^ Resources are positive aspects of work (physical, social, or organisational) that may: (1) facilitate accomplishment of goals; (2) reduce job demands and psychophysiological costs; and (3) encourage personal development. Demands are negative job characteristics (physical, social or organisational) which require continuous physical/mental effort.

Any of these factors (Table 1) can influence health and wellbeing (not necessarily associated with work). Where high demands exist, greater effort is required to maintain performance. European studies have demonstrated causality such that high demands lead to exhaustion, burnout and depression, whereas limited resources impact staff disengagement.^18^, ^19^ Conversely, adequate resources were an accurate predictor of future work engagement and organisational commitment.^19^ As such, personal resources are proposed to facilitate the achievement of work goals, stimulate personal development, and foster staff resilience.^19^ The JD‐R model is available as an organisational assessment tool to evaluate employee stress and burnout.^18^

**Table 1:** Job demands and resources

Demands	Resources	Personal resources
Centralisation	Advancement	Emotional and mental competencies
Cognitive demands	Appreciation	Extraversion
Complexity	Autonomy	Hope
Computer problems	Craftsmanship	Intrinsic motivation
Demanding contacts with patients	Financial rewards	Low neuroticism
Downsizing	Goal clarity	Need satisfaction (autonomy, belongingness, competence)
Emotional demands	Information	Optimism
Emotional dissonance	Innovative climate	Organisation‐based self‐esteem
Interpersonal conflict	Job challenge	Regulatory focus (prevention and promotion focus)
Job insecurity	Knowledge	Resilience
Negative spill‐over from family to work	Leadership	Self‐efficacy
Harassment by patients	Opportunities for professional development	Value orientation (intrinsic and extrinsic values)
Performance demands	Participation in decision making	
Physical demands	Performance feedback	
Problems planning	Positive spill‐over from family to work	
Qualitative workload	Professional pride	
Reorganisation	Procedural fairness	
Remuneration	Positive patient contacts	
Responsibility	Quality of the relationship with the supervisor	
Risks and hazards	Safety climate	
Role ambiguity	Safety routine violations	
Role conflict	Social climate	
Sexual harassment	Social support from colleagues	
Time pressure	Social support from supervisor	
Unfavourable shift work schedule	Skill utilisation	
Unfavourable work conditions	Strategic planning	
Work pressure	Supervisory coaching	
Work‐home conflict	Task variety	
Work overload	Team cohesion	
	Team harmony	
	Trust in management	

Reproduced from Schaufeli and Taris, with permission by Springer Nature and Copyright Clearance Center, February 5th 2018.^9^

### The job characteristics (JC) model

**Figure 1:**
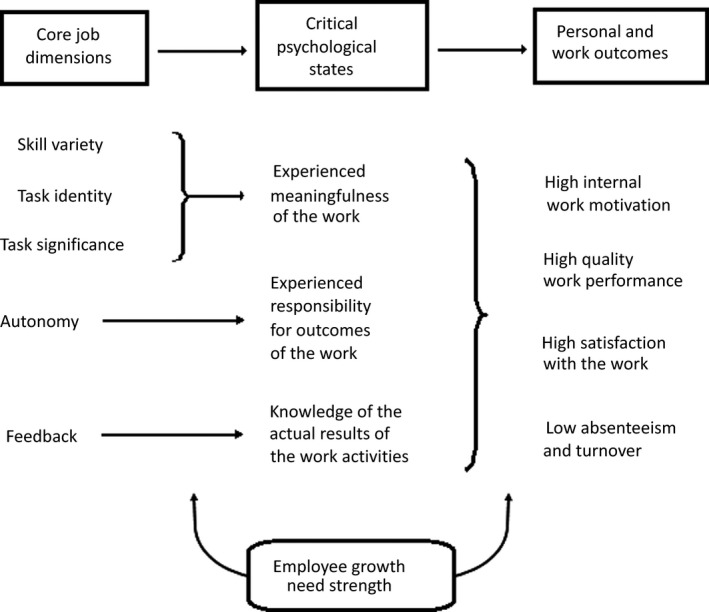
The JC model of work motivation. Reproduced from Hackman and Oldham, with permission by Elsevier and Copyright Clearance Center, February 5th 2018.^16^

The second psychological model – the JC model – considers job motivation and mental challenge.^17^ Specifically, it describes the association between job characteristics and personal motivation. Probst and Griffiths state that ‘*the extent of mental challenge is influenced by the design of the job and extent of work responsibilities in RT and this might affect retention in the profession*’.^10^ The JC model (Fig. 1) describes five factors that contribute to positive mentality; task identity, task significance, skill variety, autonomy and feedback.^17^ Addressing these factors within the workplace allows staff to contribute to personal and organisational goals. However, a failure to do so can negatively impact staff wellbeing and productivity.

Within RT practice, task identity is challenged by multidisciplinary team (MDT) role responsibility, and an inability to quantify success with unknown patient outcomes. Skill variety may be hampered by limited development opportunities and a defined scope of practice.^3^ Autonomy in decision making may be restricted to leaders – senior RTs and radiation oncologists. Further evidence correlates persistent negative feedback and poor social cohesion with dissatisfaction and demotivation.^11^ Similarly, emotional exhaustion, heavy workload, organisational constraints, conflict and dysfunctionality increase stress.^12^

RT leaders that account for mental challenge and pressure attributed to radiation‐induced errors may allow for a renewed workplace culture.^10^ POP initiatives should seek to improve staff confidence, trust, support and education to reduce stress and burnout.^12^ A number of leadership strategies will be discussed below.

## Radiotherapy Leadership Strategies

Summers and Middleton first proposed incorporating POP principles to the Australian RT workforce.^20^ The authors suggest that POP could enhance staff performance development by building upon individual strengths in addition to traditional competency measures and staff training.^20^ In support, Halkett et al.^21^ advocate for a stronger commitment from the RT leadership. The authors claim that leaders must overcome role deficiencies affecting the delivery of quality care, workplace support, working conditions and staff lifestyle.

Authentic leaders provide inspiration and a clear vision for a common goal.^22^ This is achieved by developing trust, respect, commitment, mutual understanding and a desire to succeed. Encouraging passion and teamwork can assist in developing engagement, and subsequent organisational success.^23^ Leaders should thus retire target‐driven approaches that fail to recognise individual and collaborative efforts. Instead, strategies should foster workplace relations, career progression opportunities, staff appreciation and leadership visibility. Improvement strategies may include establishing programs for mentorship, employee recognition, performance management and a social committee.^24^ Similarly, one might consider improvement to the physical environment, appealing to audio‐visual design.^25^

To facilitate a workplace culture aligned with POP, leaders should: (1) improve engagement and collaboration; (2) appeal to emotional needs; and (3) reduce job stressors.

### Improve engagement & collaboration

Staff engagement is paramount to successful change management. Appreciative inquiry is a change management strategy defined as a vision‐led group process that seeks positive development, rather than appeal to existing problems.^26^ Appreciative inquiry initiates engagement and commitment to a mutual goal. Open space meetings deliver an effective appreciative inquiry intervention to generate creative solutions – providing individuals can work collaboratively.^27^ Open space meetings allow staff to contribute to discussions, irrespective of seniority. This intervention initiates passion and motivates staff to take action on workplace issues.^27^, ^28^ Fostering passion is often overlooked by employers in lieu of outcome performance indicators.^23^ Passion cultivates enthusiasm and intention – fundamental components of change.^29^

Australian RT research recognises the need to improve communication, peer support, morale, MDT integration, conflict resolution, bullying, and career advancement processes.^5^ Overcoming these barriers to staff engagement is vital for individual and organisational growth.^20^ Possible strategies may include initiating a peer‐support group, collaborative decision‐making, counselling provision, staff recognition, debriefing, incentive programs and resilience training.^5^, ^7^, ^21^ Improvement of working conditions – staffing levels, pay, flexibility, development opportunities and department structure – may also benefit staff cohesion.

Staff collaboration requires mutual understanding and respect. Assessment of staff wellbeing and character strengths can aid in developing a collaborative workplace.^20^, ^30^, ^31^, ^32^ A wellbeing questionnaire allows individuals to understand their stress profile and organisations to analyse staff trends.^30^ Combining this tool with a character strengths assessment may improve staff integration, respect and cohesion.^31^ The Myers Briggs Type Indicator (MBTI) is one validated example to identify personality differences.^32^

### Appeal to emotional needs

As staff spend considerable time at work, the need for workplace positivity is ever‐present. Positive emotions of hope, joy, gratitude, interest, aspiration and pride can help to shape workplace culture, foster mental health and increase life satisfaction.^33^ Promotion of positive emotions is contagious, but reliant upon staff commitment.^34^ To promote positive emotions, leaders should sincerely and frequently recognise efforts, provide support, promote work‐life balance, and encourage confidence and productivity.^7^, ^20^, ^21^ However, positive emotions should not occur sporadically, but rather contribute to a positive whole‐life experience.^35^

In pursuing the ‘*good life*’, individuals seek activities that are intrinsically satisfying and motivating.^33^ McClelland's trichotomy of needs theory proposes that motivation stems from three needs – affiliation, power and achievement.^36^ Understanding individual's gravitation to these needs can help leaders to foster an environment that supports personal attributes.

### Reduce job stressors

Stress results from increased demands that exceed an individual's ability to cope.^35^ Stress can influence reduced collaboration, poor performance and increased error.^34^ Staff resilience training may assist in overcoming workplace stressors.^5^ This strategy is enhanced by a supportive and trustworthy culture.^33^ Investment in social capital (resilience education), likely increase staff satisfaction and performance. Social capital interventions are best developed in collaboration with role models within the workplace and community.^35^

Stress is often considered a precursor to burnout – a condition comprising emotional exhaustion, depersonalisation and reduced capacity to function effectively.^28^ Stress is also associated with high attrition.^35^ Predictors of burnout in healthcare include increased work hours, monotonous tasks, elevated expectations, negative patient interactions, financial burden and poor perceptions of workplace support.^3^, ^28^ In avoiding stress and burnout, one must assess workplace stressors; accounting for personal vulnerability (external stressors) and workplace support mechanisms.^28^ Improvement strategies should address job characteristics, leadership style and specific job stressors.^37^ This may entail improved career progression opportunities, education support, development activities, leadership training, communication pathways and personalised roles.

Current research reports higher burnout rates amongst Australian RTs than previous international studies.^6^, ^7^, ^38^ Utilising the Maslach Burnout Inventory (MBI), RTs reported considerable emotional exhaustion, depersonalisation and reduced personal accomplishment.^6^, ^7^ Furthermore, public hospital staff reported higher emotional exhaustion than colleagues in private practice.^7^ In either sector, elevated burnout was attributed to environmental factors, rather than patient interactions. As such, leaders must consider the impact of burnout and commit to appropriate monitoring/prevention.

A greater risk of burnout has been associated with an elevated patient workload, refusal to take leave, and declined participation in communication skills training.^6^ Hansen and Girgis^38^ propose a single‐item burnout screening tool to measure prevalence more efficiently than the MBI. The single‐item tool is a reliable predictor of outcomes and is highly correlated with the MBI – suggesting it could complement a regular staff wellbeing survey. Combining this tool with a performance development plan could allow leaders to implement strategies to improve workplace culture, engagement and patient outcomes.^20^

Burnout and stress are attributed to high attrition and absenteeism.^7^ Research suggests that stressors result from poor working conditions: staff shortages, increased workload, interpersonal challenges, unsupported technological advancement and unreasonable demands.^1^, ^21^ However, the most prevalent stressor is dissatisfaction in career progression opportunities.^1^, ^3^, ^5^, ^7^, ^39^ Leaders should explore opportunities for advanced practice,^3^, ^5^, ^7^ subspecialisation,^3^, ^5^ research,^39^ education^7^ and relinquishment of non‐core roles.^1^ This may consider job redesign, greater flexibility and leadership transparency.^7^

## Conclusion

Several psychological models have been discussed in the Australian RT context. The JD‐R model describes stress as influenced by competing resources and demands. Similarly, the JC model explores the factors that contribute to workplace mental challenge. Both models apply to RTs and encourage regulation of stressors impacting burnout and attrition. To overcome such unfavourable outcomes, POP offers an alternative to traditional leadership methodology and inherent workplace culture. A leadership emphasising workplace success will encourage fulfilment of emotional needs and staff engagement to counteract elevated stress, burnout and attrition. The success of this approach is strongly correlated with a respectful and methodical implementation. A number of specific strategies are provided to aid improvement of the Australian RT workplace culture. However, one must note the absence of RT literature to ratify the efficacy of POP implementation. Further research is encouraged to quantify the benefit of POP leadership strategies to reduce burnout and attrition, in favour of a consolidated Australian RT workforce.

## Conflict of Interest

The authors declare that they have no competing interests.
